# Dynamics of Connexin 43 Down Modulation in Human Articular Chondrocytes Stimulated by Tumor Necrosis Factor Alpha

**DOI:** 10.3390/ijms23105575

**Published:** 2022-05-16

**Authors:** Elena Della Morte, Stefania Niada, Chiara Giannasi, Luigi Zagra, Anna Teresa Brini

**Affiliations:** 1 IRCCS Istituto Ortopedico Galeazzi, 20161 Milan, Italy; elena.dellamorte@grupposandonato.it (E.D.M.); chiara.giannasi@unimi.it (C.G.); luigi.zagra@fastwebnet.it (L.Z.); anna.brini@unimi.it (A.T.B.); 2 Department of Biomedical, Surgical and Dental Sciences, University of Milan, 20157 Milan, Italy

**Keywords:** Connexin 43, articular chondrocytes, TNFα, proteasome, TGF-β1

## Abstract

Connexin 43 (Cx43) exerts pivotal functions in articular chondrocytes (CH). It is involved in the communication among cells and between cells and the extracellular environment, and it contributes to the maintenance of the correct cell phenotype. The pro-inflammatory cytokine TNFα induces a reduction in Cx43 expression in CH. Here, we studied the dynamics of this decrease in expression. We evaluated Cx43 protein and gene expression and the involvement of C-terminal domain (CTD) cleavage and proteasomal degradation. Treatments able to counteract TNFα action were also examined, together with Gap Junction (GJ) functionality and Cx43 localization. TNFα induced a significant reduction in Cx43 expression already at day 1, and the down modulation reached a peak at day 3 (−46%). The decrease was linked to neither gene expression modulation nor CTD cleavage. Differently, the proteasome inhibitor MG132 reverted TNFα effect, indicating the involvement of proteasomal degradation in Cx43 reduction. In addition, the co-treatment with the anabolic factor TGF-β1 restored Cx43 levels. Cx43 decrease occurred both at the membrane level, where it partially influenced GJ communication, and in the nucleus. In conclusion, TNFα induced a rapid and lasting reduction in Cx43 expression mostly via the proteasome. The down modulation could be reverted by cartilage-protective factors such as MG132 and TGF-β1. These findings suggest a possible involvement of Cx43 perturbation during joint inflammation.

## 1. Introduction

Connexins are transmembrane proteins highly expressed and conserved in chordates [1]. They have four transmembrane domains, one cytoplasmic and two extracellular loops, a cytoplasmic N-terminal domain (NTD) and a cytoplasmic C-terminal domain (CTD), which represents the most variable region among connexins in terms of length and sequence. Connexins are largely known for their role in direct communication between the cytosols of adjacent cells through gap junctions (GJ). These intercellular connections, that permit the flow of ions and molecules smaller than 1 kDa, are made up of hundreds to thousands of connexons (or connexin hemichannels), that are, in turn, constituted by the assembly of six connexins, docked with the hemichannels of contiguous cells [1]. In addition to intercellular communication via GJ, single-cell connexin hemichannels allow the exchange of small molecules between the cell cytosol and the extracellular environment. Moreover, connexins also interact with different signaling pathways, thus influencing a multitude of cellular processes.

Out of the 21 human connexins, Connexin 43 (Cx43) is the most widely expressed and best characterized. It exerts important functions spanning from intercellular communication to the modulation of cell growth and differentiation [2]. It possesses a particularly complex C terminal domain (CTD) of about 150 amino acids by which it interacts with proteins of the cytoskeleton (e.g., Tubulin, ZO-1, Drebin) and with regulators of hemichannel activity (e.g., integrin-α5), cell growth, differentiation and migration, among which include β-catenin, Cyclin E and β-arrestin [3].

Cx43 fine-tuning is essential for the development, structure, and function of multiple tissues, including articular cartilage. Cx43 is located at the edges of lacunae, on the membranes between the cytoplasms of contiguous CH, and on cellular projections [4]. Through Cx43 channels, chondrocytes (CH) exchange a multitude of molecules with the extracellular environment and with adjacent and distant cells. Cx43 is also involved in the maintenance of chondrocytes phenotype by interacting with a wide repertoire of intracellular proteins mainly through its CTD. Indeed, in transgenic mice, the expression of a truncated form of Cx43 lacking the last 125 amino acids (Cx43K258stop) leads to an alteration of cartilage structure with a reduction in proteoglycans and abnormal CH phenotype characterized by increased proliferation and reduction in collagen II expression [5].

The correlation between Cx43 and the maintenance of a correct CH phenotype was also suggested in our previous work. In particular, the treatment with the pro-inflammatory cytokine tumor necrosis factor alpha (TNFα) induced both the shift toward a hypertrophic phenotype [6,7] and a significant down modulation of Cx43 [7].

TNFα is one of the major catabolic factors in inflamed cartilage. In addition to its direct action on CH, it also induces the release of MMPs from synovial fibroblasts, which results in further cartilage destruction [8]. In opposition to the catabolic action of TNFα, TGF-β1 exerts a key function in regulating the differentiation, proliferation, terminal differentiation, and maintenance of articular chondrocytes phenotype [9,10]. In addition, its positive effect has been also shown in the OA context. Indeed, arthritic patients treated with TGF-β1-expressing CH displayed an improved clinical outcome [11]. Interestingly, this anabolic factor also induces the upregulation of Cx43 expression in CH [12,13].

Considering the importance of Cx43 in chondrocytes and cartilage physiology and the presence of high levels of TNFα in multiple joint-affecting conditions, we decided to deepen our knowledge on the TNFα-mediated reduction in Cx43. We aimed at (i) evaluating the kinetics of TNFα-induced Cx43 reduction in primary articular chondrocytes, (ii) investigating the mechanism underlying this modulation, (iii) identifying treatments able to counteract this effect and (iv) hypothesizing consequences of this modulation.

## 2. Results and Discussion

In this study, we explored the modulation of Cx43 in TNFα-stimulated chondrocytes (CH) in order to shed light on the regulation of this protein with fundamental importance in the physiology of CH.

**Figure 1:**
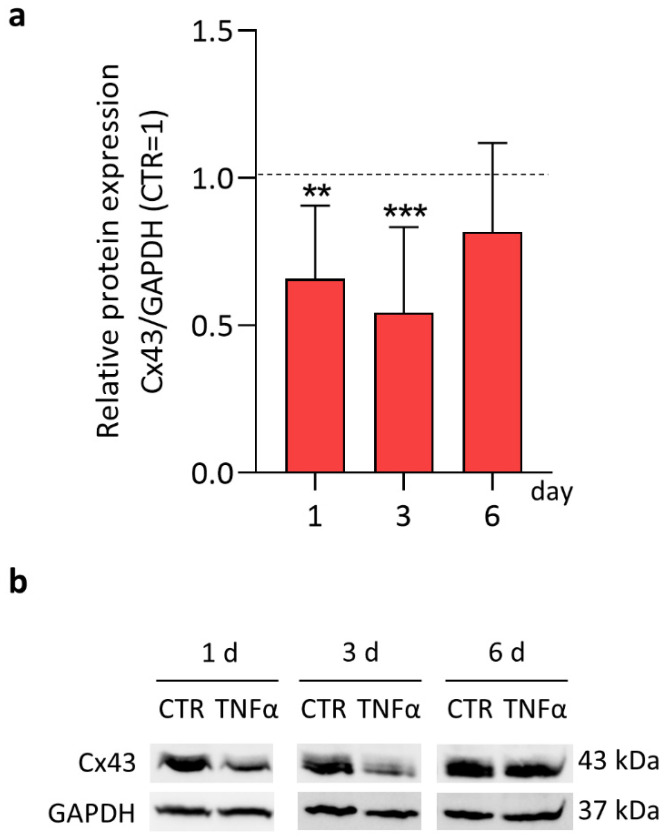
Expression of Cx43 in TNFα-stimulated CH at day 1, 3 and 6 analyzed by Western blot. (**a**) Specific bands were quantified through Image Lab Software v 6.1 (Bio-Rad, Milan, Italy), and data (day 1, *n* = 7; day 3–6, *n* = 11) were normalized on GAPDH and expressed as relative values (CTR = 1). Statistical analysis was performed by paired *t* test. Significance vs. appropriate CTR for each time point are shown as ** *p* < 0.01 and *** *p* < 0.001. (**b**) Representative immunoblots are shown. CTR: control (untreated CH).

In our setting, articular chondrocytes were isolated from macroscopically healthy cartilage of osteoarthritic (OA) patients [6,7,14]. Cx43 was highly expressed, as previously observed in CH derived from both healthy and OA subjects [15]. The effect of 10 ng/mL TNFα on Cx43 expression was monitored at days 1, 3 and 6. Cx43 expression was significantly reduced by 34% at day 1 compared to control (−34 ± 25%) (Figure 1a, left bar, Figure 1b, left panel) and showed a reduction by almost half on day 3 (−46 ± 29%) (Figure 1a, middle bar, Figure 1b, middle panel). This confirms the effect of the pro-inflammatory cytokine on CH [7] and other cell types such as keratinocytes [16], human corneal fibroblasts [17,18] and astrocytes [19,20]. After 6 days, 25% of populations (4 out of 11) was less responsive to TNFα action. Consequently, the average effect of the pro-inflammatory cytokine appeared decreased (−18 ± 30% vs. CTR) (Figure 1a). The donor-related variability is the most likely cause of the differences we observed. Future studies might focus on donor variables (e.g., age, sex, BMI, OA-grade) associated to the different CH response at extended time-points.

We then investigated mechanism/s possibly involved in TNFα-mediated Cx43 effect. At first, we evaluated whether a decrease in *GJA*1 (Gap Junction Protein Alpha 1) transcription could be involved, as previously observed in keratinocytes [16] and liver epithelial cells [21]. However, TNFα induced no modulation of *GJA*1 mRNA levels in CH, neither at day 1 nor at day 3 (Supplementary Figure S1).

**Figure 2:**
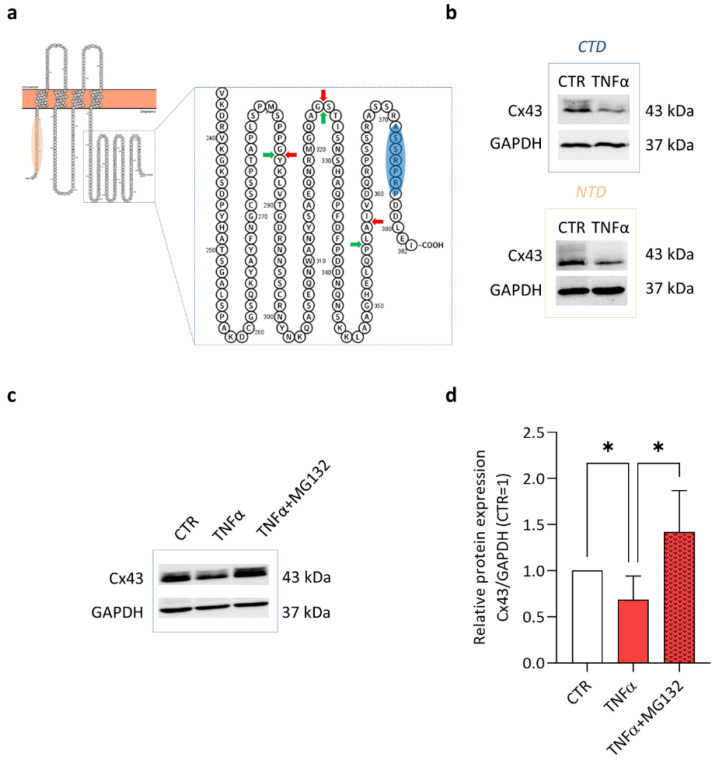
**Cx43 cleavage and proteasomal degradation in TNFα-treated CH.** (**a**) Schematic amino acid sequence of Cx43 (https://wlab.ethz.ch/protter/# (accessed on 15 March 2021)). The major 3 MMP-3 and -13 cleavage sites at the CTD, predicted by in silico analysis (https://www.dmbr.ugent.be/prx/bioit2-public/SitePrediction/ (accessed on 15 March 2021)), are shown as red and green arrows, respectively. The area surrounding Arg376, the recognized epitope of the CTD antibody, is highlighted in blue, while the one at the NTD is in orange. (**b**) Representative immunoblot of CTR and TNFα-treated CH at day 3 with specific band for Cx43 revealed by Ab raised against epitope at either CTD or NTD. (**c**,**d**) Western blot analysis of Cx43 expression in CH pre-treated for 1 h with MG-132 and then stimulated with TNFα for 24 h. (**c**) Representative immunoblot is shown. (**d**) Quantification of Cx43 expression. Data (*n* = 7 independent experiments) were normalized on GAPDH and expressed as relative values (CTR = 1). Statistical analysis was performed by one-way analysis of variance (ANOVA) using Tukey’s post hoc test. Significance is shown as * *p* < 0.05. CTD: C-terminal domain, CTR: control (untreated), Cx43: Connexin 43, GAPDH, Glyceraldehyde-3-Phosphate Dehydrogenase, MMP: matrix metalloproteinase, NTD: N-terminal domain, TNFα: tumor necrosis factor alpha.

Next, we evaluated Cx43 CTD cleavage. This hypothesis sounded particularly intriguing, since the pro-inflammatory cytokine TNFα is known to induce an evident increase in MMP expression and activation in CH [6,7,22]. Here, we evaluated MMP-3 and -13 expression (Supplementary Figure S2), while a complete evaluation of gene/protein expression and MMP release and activity in the same experimental setting (human articular chondrocytes treated with 10 ng/mL TNFα) was previously performed [6]. These proteases can be involved in Cx43 CTD cleavage, thus contributing to inflammatory response [23]. In silico analysis indicated multiple cleavage sites recognized by MMP3 and MMP13 (Figure 2a red and green arrows, respectively) at the CTD.

If CTD cleavage had occurred in our setting, we should have observed no difference in Cx43 expression by using an antibody raised against the N-terminal domain. Moreover, CTD fragments should have been detectable by Western blot. On the contrary, the same reductions were observed with both antibodies (Figure 1, Figure 2b and Supplementary Figure S3), and smaller molecular weight bands were never observed (e.g., in Supplementary Figures S4 and S7). This indicated that TNFα treatment does not lead to Cx43 cleavage in our in vitro model.

At last, considering that the proteasomal degradation has been previously associated to the reduction in Cx43 induced by TNFα [17,20], we also investigated this aspect. Cells were treated for 1 h with the proteasome inhibitor MG132 (1 µM) before stimulating with TNFα for 24 h. The TNFα-induced downregulation of Cx43 (Figure 2c,d) was inhibited by MG132, thus suggesting that the proteasomal degradation was one of the main causes of Cx43 reduction.

**Figure 3:**
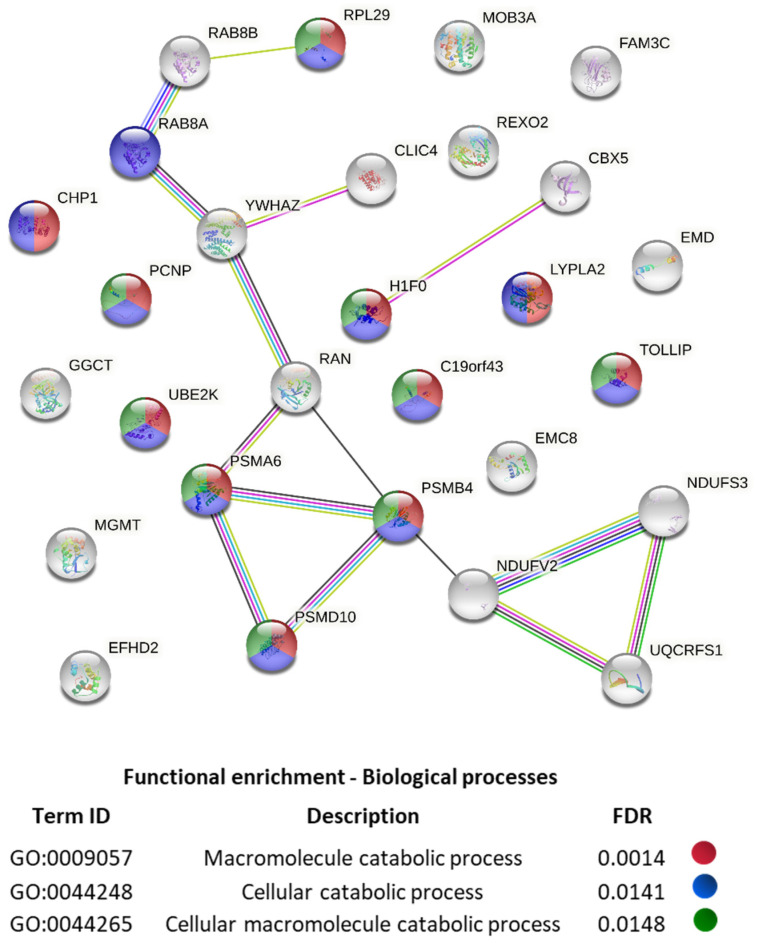
STRING analysis of TNFα-induced factors (default settings; edges represents protein-protein associations, nodes are filled if 3D structure is known). Protein–protein interactions and top 3 biological processes are shown. The colors in the circles represent the three biological processes.

Interestingly, the TNFα stimulation also induced the increase in the expression of proteins linked to protein catabolism. In particular, we observed the intensification of a band around 25 KDa in the SDS-PAGE of TNFα-treated CH (data not shown). Mass spectrometry analysis of this SDS-PAGE area revealed 28 proteins expressed only by TNFα-treated CH (Figure 3, Supplementary Table S1). A functional association analysis (STRING [24]) highlighted a significant enrichment in factors involved in macromolecule catabolism (Figure 3, Supplementary Table S2).

Among them, we recognized two components of the 20 Score proteasome complex (PSMA6 and PSMB4), which is a component of the proteasome regulatory system (PSMD10) and of the ubiquitin-conjugating enzyme E2 K (UBE2K). Although this is just a partial analysis, it suggests that the ubiquitin–proteasome system might be activated in TNF-stimulated CH, leading most likely to an increase in Cx43 degradation, as previously observed in spinal astrocytes [20].

The association between TNFα and proteasome activation in CH has been recently shown by Deng et al., 2018. TNFα induced poly-ubiquitination and degradation of the protein YAP, suggesting that this process can be involved in OA development as well.

Other in vitro and in vivo evidence supports the contribution of proteasome activity to cartilage damage and OA progression [25,26,27,28].

Accordingly, MG132 has been shown to protect cartilage against the effects of inflammatory mediators by regulating MMP [26], antioxidant defense [27], and cartilage markers [28].

**Figure 4:**
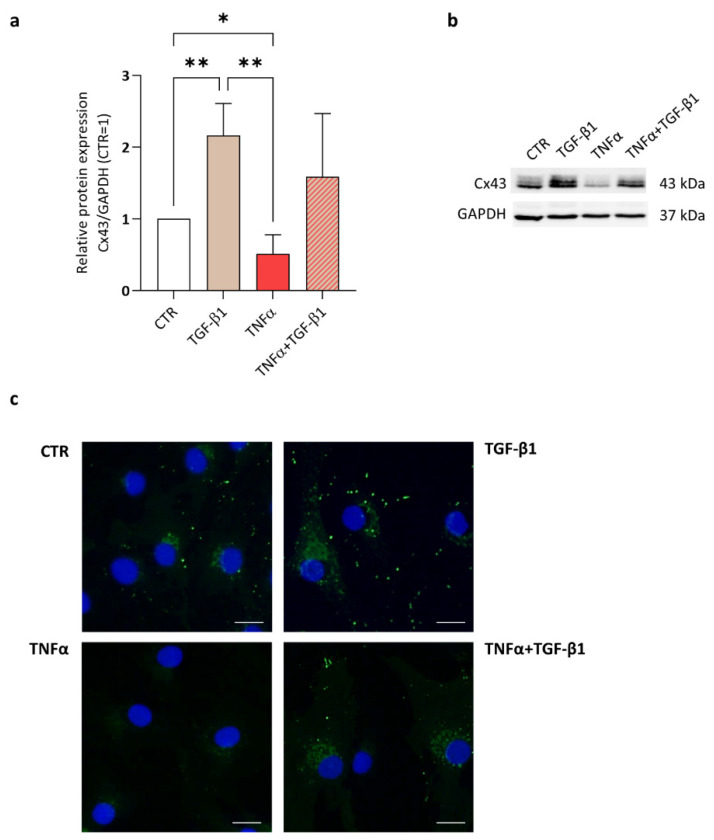
The effect of TNFα and/or TGF-β1 on Cx43 expression in CH. (**a**) Quantification of Cx43 expression, analyzed by Western blot, in CH treated with TNFα or/and TGF-β1 at day 3. Data (*n* = 6 independent experiments) were normalized on GAPDH and expressed as relative values (CTR = 1). Statistical analysis was performed by one-way analysis of variance (ANOVA) using Tukey’s post hoc test. Significance are shown as * *p* < 0.05 ** *p* < 0.01. (**b**) Representative immunoblot. (**c**) Connexin 43 expression by immunofluorescence (green signal) in CH either untreated or treated for 3 days with TGF-β1, TNFα and TNFα + TGF-β1. Nuclei are counterstained with DAPI and scale bars: 20 µm. CTR: control (untreated), TGF-β1, transforming growth factor beta 1, TNFα: tumor necrosis factor alpha.

Furthermore, we investigated if other cartilage-protective factors could prevent Cx43 reduction by TNFα. Anabolic factors, such as IGF, HGF, PDGF and TGF, have been shown to increase Cx43 expression in different tissues and cell types [29,30,31,32,33]. Among them, TGF-β1 action has been previously described in CH [12]. Here, we evaluated whether this anabolic cytokine could counteract TNFα effect. The treatment with TGF-β1 (5 ng/mL) for 3 days increased the expression of Cx43 (+116% vs. CTR). Interestingly, this multifunctional cytokine counteracted the TNFα-induced reduction in Cx43 (+209% vs. TNFα) (Figure 4a,b). These effects were less evident at day 6 (Supplementary Figure S5). These results were confirmed by immunofluorescence analysis; both the classical punctiform Cx43 signal and the staining near the nucleus (Figure 4c) were modified by the treatments, suggesting that Cx43 modulation occurs at both the membrane level and in other compartments.

**Figure 5:**
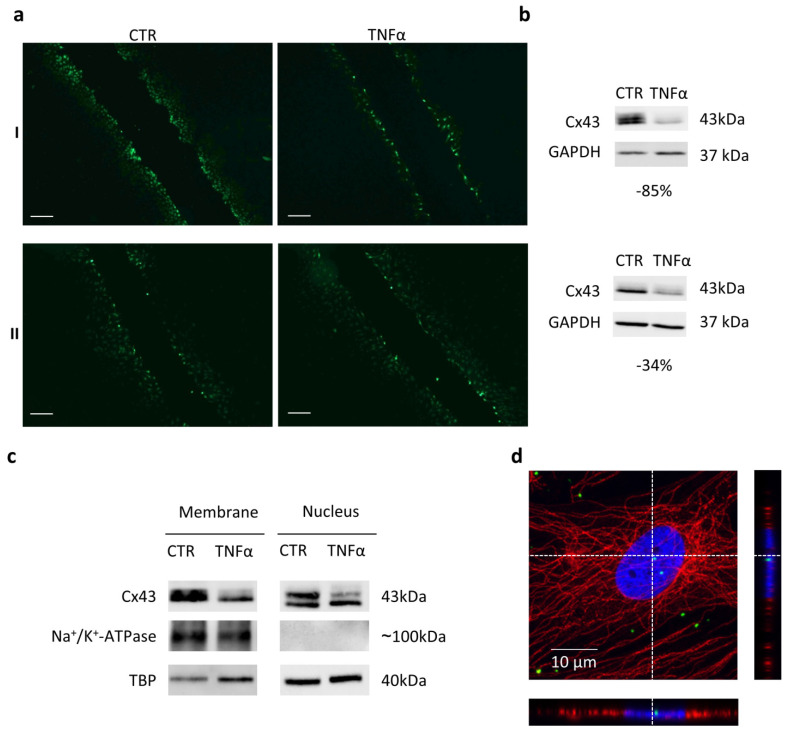
(**a**) Illustrative images of scrape loading/dye transfer (SL/DT) assay in CTR (**on the left**) and TNFα-treated (**on the right**) CH, at day 3. Scale bars: 200 µm. (**b**) Immunoblot of each experiment is reported. The percentage of decrease in Cx43 in TNFα-treated CH compared to CTR is shown. (**c**) Western blot analysis of Cx43 expression and modulation in membrane and soluble nuclear fractions of CTR and TNFα-treated CH at day 3. Na^+^/K^+^-ATPase and TBP are shown as control for the membrane and nucleus fraction, respectively. (**d**) Laser scanning confocal microscopy of CH treated with TNFα for 3 days. Cx43 and β-Tubulin were revealed with an Alexa Fluor^®^ 488 (Cx43) and 568 (β-tubulin) conjugated antibody (green and red respectively), nuclei were stained with DAPI (blue) (magnification 63×). The scale bar indicates 10 μm, and the orthogonal views were obtained by Fiji software (ImageJ 1.51). CTR: control (untreated), Cx43: Connexin 43, GAPDH, Glyceraldehyde-3-Phosphate Dehydrogenase, Na^+^/Ka^+^ ATP-ase: sodium–potassium ATPase, TBP: TATA-bindin protein, TNFα: tumor necrosis factor alpha.

Since Cx43 displays an important role in CH communication, through GJ, we investigated if TNFα-induced reduction in Cx43 affected intercellular communication by a scrape loading assay based on Lucifer yellow (LY) dye transfer into contiguous cells [34]. We analyzed four CH populations. From these analyses, it appeared that differences between treated and non-stimulated CH become detectable only when the impairment of Cx43 was evident. Particularly, the analysis of fluorescent images showed a reduction in dye penetration (Figure 5(aI) and Supplementary Figure S6(aIII), Supplementary Table S3) when the TNFα effect on Cx43 was marked (Figure 5(bI) and Supplementary Figure S6(bIII), Supplementary Table S3). On the contrary, no major difference in the dye transfer was observed (Figure 5(aII) and Supplementary Figure S6(aIV), Supplementary Table S3) when the pro-inflammatory cytokine action on Cx43 was less pronounced (Figure 5(bII) and Supplementary Figure S6(bIV), Supplementary Table S3).

This could suggest that GJ impairment occurs only when Cx43 expression is strongly affected by TNF-α treatment. Since we observed Cx43 reduction at the membrane level (Figure 5c), we can speculate that this decrease influences connexin hemichannel activity rather than intercellular communication.

This is an interesting hypothesis considering that in articular cartilage, CH exposes most of the cell borders, rich in Cx43 hemichannels, to the extracellular matrix [4]. Of note, the importance of connexin hemichannel rather than intercellular communication, for appropriate CH differentiation and physiology, has been previously shown [35].

Nevertheless, another possible explanation may rely on a lower specificity and sensibility of the LY imaging assay in comparison to Western blot.

Cx43 was not reduced only at the cell membrane level. Indeed, in our setting, Cx43 appeared enriched in the soluble nuclear fraction (Figure 5c and Figure S7), and confocal microscopy analysis showed that this protein co-localized in the nuclei with DAPI counterstain (Figure 5d). Consistently, the presence of full-length Cx43 or CTD fragments in the nucleus has been described and associated to this connexin effect on cell growth and differentiation [2,3,36]. A proteomic analysis of Cx43 in articular chondrocytes highlighted the involvement of Cx43 in transcription processes, such as RNA splicing, processing, export and translation, post-transcriptional regulation and mRNA processing [37]. Our data show that TNFα induced a decrease in Cx43 in the nuclear compartment as well. It is plausible that the alteration of Cx43 levels in the nucleus might contribute to some of the TNFα effects on CH, such as the hypertrophic shift and the increase in cell proliferation [6]. Interestingly, the above-mentioned proteomic analysis showed an enrichment of Cx43 interactors related to regulation of transcription in healthy CH compared with OA ones [37]. Further investigations are required to shed light on this complex aspect.

Up until now, the contribution of Cx43 in joint diseases such as osteoarthritis and rheumatoid arthritis (RA) has been controversial. On one hand, Cx43 is highly expressed in healthy chondrocytes where it allows both cell to cell communication and the exchange between cells and the extracellular matrix and contributes to the maintenance of CH phenotype [4,5]. On the other hand, some studies show that Cx43 levels are increased in OA- and RA-affected patients. However, whether these increased levels imply a connection between Cx43 and the pathologies or it represents a compensatory mechanism is still under debate [1].

Since TNFα levels are particularly elevated after acute injury rather than during chronic disease [38,39,40], it can be hypothesized that in these contexts, the pro-inflammatory cytokine-induced reduction in Cx43 acts the most. Until now, there have been no studies on this aspect. Future investigations might shed light on Cx43 regulation and involvement at the early phase of joint injury.

In this context, investigations on the effect of TNFα in chondrocytes derived from healthy subjects will be useful to understand whether a reduction in Cx43 participates in the initial steps of joint damage.

In conclusion, with this study, we showed that TNFα induces a rapid and lasting reduction in Cx43 in articular chondrocytes that is mainly due to its degradation via the proteasome. The proteasome inhibitor MG132 as well as the anabolic factor TGFβ1, both known as cartilage-protective factors, counteract this modulation. In our in vitro model, the decrease in Cx43 occurs at both the membrane and nuclear level, suggesting possible alterations in multiple aspects of CH physiology. Our data might indicate that TNFα effects on CH and cartilage could also be linked to Cx43 reduction, thus implying an involvement of this protein perturbation during joint inflammation.

## 3. Materials and Methods

Unless otherwise specified, reagents were purchased from Sigma-Aldrich (St. Louis, MO, USA).

### 3.1. Cell Cultures

Primary human articular chondrocytes (CH) were obtained from waste tissue collected at IRCCS Istituto Ortopedico Galeazzi upon Institutional Review Board approval. In detail, CH were isolated from the femoral heads of 17 patients (7 males, 10 females) who underwent total hip replacement. Only the areas of macroscopically healthy cartilage (white, shiny, elastic, and firm) were harvested. The areas characterized by irregular surface, discoloration or softening were not collected in order to exclude any experimental bias linked to the use of strongly compromised cartilage [6,7]. Cartilage specimens were then cut with a scalpel and digested overnight at 37 °C with 1.5 mg/mL Collagenase type II (Worthington Biochemical Corporation, Lakewood, NJ, USA). The next day, collagenase solution was inactivated with FBS-containing culture medium, and the resulting cells suspension was filtrated with a 100 µm cell strainer. Cells were then counted and plated at the density of 10^4^/cm^2^ in a complete medium consisting of high glucose DMEM supplemented with 10% FBS (Euroclone, Pero, Italy), 2 mM glutamine, 50 µg/mL streptomycin, 50 U/mL penicillin and sodium pyruvate 110 µg/mL.

### 3.2. Treatments

For all experiments, CH were used at first passage and seeded at the density of 10^4^/cm^2^. Cells were cultured in complete medium until they reached the confluence; then, they were shifted in a complete medium with 1% FBS and treated with either 10 ng/mL TNFα, to mimic the inflammatory environment, or with 5 ng/mL TGFβ, acting as an anabolic factor, for 1, 3, 6 days without any other media change. In specific experimental settings, the inhibitor of the proteasome MG132 at 1 µM, was administered for 1 h prior to TNFα treatment.

### 3.3. Cell Lysates

CH were lysed in 50 mM Tris-HCl (pH 7.5), 150 mM NaCl, 1% NP-40 and 0.1% SDS with added protease inhibitor cocktail (PIC) and 2 mM PMSF. After an incubation in ice for 30 min, lysates were centrifuged at 14,000× *g* for 10 min at 4 °C. Supernatants were then stored at −20 °C until use. Protein content was quantified through BCA assay (Thermo Fisher Scientific, Waltham, MA, USA), and 10 µg of proteins for each sample was analyzed by 10% SDS-PAGE and Western blotting as described below.

### 3.4. Subcellular Fractionation

Subcellular protein fractionation was performed using a Subcellular Protein Fractionation Kit (Thermo Fisher Scientific, Waltham, MA, USA). Confluent CH were harvested with trypsin–EDTA and then lysed and centrifuged following manufacturer’s protocol. Then, 5 µg of protein content of each sample was analyzed by 10% SDS-PAGE and Western blotting as described in the following paragraph. Na^+^/K^+^-ATPase and TBP were used as control for the enrichment of the membrane and nuclear fraction, respectively.

### 3.5. Western Blotting

Samples were analyzed by 10% SDS-PAGE and Western blotting, using standard protocols. Primary antibodies were incubated ON at 4 °C: rabbit anti-Connexin 43 C-terminal (#3512, Cell Signaling, Danvers, MA, USA, 1:1000 diluted), mouse anti-Connexin 43 N-terminal (MABT903, 1:250 diluted), mouse anti-TATA binding protein TBP (ab51841 Abcam, Cambridge, UK, 0.1 µg/µL, 1:1000 diluted), rabbit anti-Sodium Potassium ATPase antibody (ab76020, Abcam, Cambridge, UK, 1:100,000 diluted) and goat anti-GAPDH (sc-20357, Santa Cruz Biotechnology, CA, USA, 0.1 µg/µL, 1:1000 diluted). Specific signals were revealed after the incubation with the appropriate secondary antibodies conjugated to horseradish peroxidase (Rabbit IgG Secondary antibody, Thermo Fisher Scientific, Waltham, MA, USA, dilution 1:6000; Mouse IgG Secondary Antibody, Thermo Fisher Scientific, Waltham, MA, USA, dilution 1:6000; Goat IgG Secondary Antibody, Santa Cruz Biotechnology, CA, USA; 0.1 μg/μL, 1:6000 diluted) followed by detection with ECL Westar Supernova (Cyanagen, Bologna, Italy). Signals were acquired by a Chemidoc Imaging System, and densitometry was quantified through Image Lab Software (Bio-Rad, Milan, Italy). In order to normalize the expression of target proteins, the band intensity of each sample was divided by the intensity of the appropriate loading control (GAPDH or TBP or Na^+^/K^+^-ATPase). Data were expressed as a ratio on the control lane of each set (CTR = 1).

### 3.6. Gene Expression Analysis

Next, 24 and 72 h after the treatments, CH were lysed and RNA was extracted using an RNeasy Micro kit (Qiagen, Hilden, Germany). DNase I treatment (15 min) was performed to eliminate genomic contamination. cDNA was synthetized with the High-Capacity Reverse-Transcription Kit according to the manufacturer’s protocol (Thermo Fisher Scientific, Waltham, MA, USA). The expression of Cx43 and the housekeeping TBP were quantified by RT-qPCR using TaqMan technology (*GJA1*: Hs00748445_s1; *TBP*: hs00427600_m1) at the StepOne Plus Applied Biosystem apparatus (LifeTechnologies, Carlsband, CA, USA). Data were analyzed with the 2^−ΔΔCt^ method.

### 3.7. Scrape Loading Assay

After 3 days of treatments, CH were rinsed three times with phosphate buffer saline added with 0.1 mM CaCl_2_ and 1 mM MgCl_2_(CaMg-PBS) and then incubated with a solution of Lucifer Yellow (1 mg/mL Lucifer Yellow CH dilithium salt). For every well, 3 scrapes were made with a tip in order to allow the dye to enter the cells. The plates were incubated for 5 min in the dark at room temperature. Then, wells were rinsed 3 times with CaMg-PBS, and cells were fixed with 10% formalin. Fluorescence images were captured by wide-field fluorescence microscopy BX51 (Olympus, Tokyo, Japan). The distance crossed by the dye from the scrape line was measured using Fiji software. For every image, 10 measurements were taken.

### 3.8. Immunofluorescence

CH were seeded on 1 cm diameter glass coverslips at a density of 10^4^ cells/cm^2^. At confluence, cells were treated with 10 ng/mL TNFα, 5 ng/mL TGF-β1 or a combination of the two treatments. After 3 days, samples were fixed in 4% paraformaldehyde, permeabilized with 0.1% Triton X-100 and incubated overnight at 4 °C with the antibody raised against Cx43 C-terminal (#3512, Cell Signaling, Danvers, MA, USA, 1:100 diluted). Specific binding was revealed with a secondary antibody conjugated to AlexaFluor 488 (A-11001, Invitrogen, Waltham, MA, USA, 1:2000 diluted), and coverslips were mounted with ProLong™ Diamond Antifade Mountant with DAPI (Thermo Fisher Scientific, Waltham, MA, USA). Samples were analyzed by wide-field fluorescence microscopy (BX51, Olympus). For confocal imaging, a double staining for Cx43 (primary antibody #3512, Cell Signaling, Danvers, MA, USA, 1:100 diluted and secondary antibody conjugated to AlexaFluor 488, Ab150073 Abcam, Cambridge, UK, 1:1000 diluted) and β tubulin (primary antibody T7815 1:500 diluted and secondary antibody conjugated to AlexaFluor 488, Ab175473 Abcam, Cambridge, UK, 1:1000 diluted) was performed following standard procedures. Samples were analyzed by the confocal laser scanning microscope TCS SP8 (Leica Microsystems CMS GmbH, Wetzlar, Germany). Images were acquired with a 63× objective and analyzed using Fiji software (ImageJ 1.51).

### 3.9. nLC-MS/MS Analysis for Protein Identification

Since a specific band in CH treated with TNFα was always revealed by Ponceau-S staining, cell lysates from a representative population were tested by nano-scale liquid chromatography tandem mass spectrometry (nLC-MS/MS). In detail, 20 µg of TNFα-treated CH and respective control cells were separated by SDS page using 4–15% precast polyacrylamide gel (Bio-Rad, Milan, Italy) following standard protocols. Gels were then stained with Coomassie dye and, both in CTR and TNF lanes, an area corresponding to the band appearing after TNFα treatment (about 25 kDa) was excised with a scalpel. For protein identification, bands were in-gel digested with trypsin and analyzed by nLC-MS/MS by a MS facility (Proteomics and Metabolomics Facility, ProMeFa, HSR San Raffaele Scientific Institute, Milan, Italy).

### 3.10. Statistical Analysis

Statistical analysis was performed by a paired *t*-test or one-way analysis of variance (ANOVA) using Tukey’s post hoc test. All the analyses were performed using Prism 9 (GraphPad Software, La Jolla, CA, USA). Data are expressed as mean ± SD and differences were considered significant at *p* ≤ 0.05.

## Data Availability

Raw data will be available in a suitable platform upon article publication.

## Supplementary Materials

The following are available online at https://www.mdpi.com/article/10.3390/ijms23105575/s1.
