# Supplementary material for: Dynamics of Connexin 43 Down Modulation in Human Articular Chondrocytes Stimulated by Tumor Necrosis Factor Alpha
Source: Int J Mol Sci. 2022 May 16;23(10):5575. doi: 10.3390/ijms23105575 (PMC9142923; doi:10.3390/ijms23105575)
Supplement: Supplementary file 1 [file ijms-23-05575-s001.zip › Supplementary Figures S1-S7.pdf]

## Supplementary figure legends

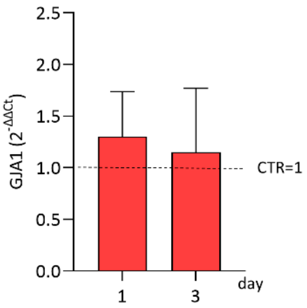


**Supplementary Figure S1.** Gene expression of GJA1 in TNFα-stimulated CH at day 1 and 3 analyzed by real-time PCR. Data (*n* ene expression of GJAiments) are expressed as $2^{-ents}$ (TBP was used as a housekeeping gene).

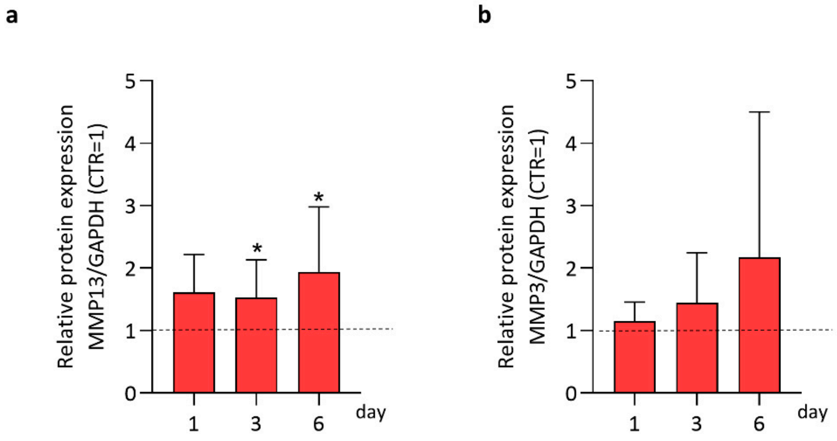


**Supplementary Figure S2**. Effect of TNFα on MMPs protein expression in CH. Quantification of the expression of MMP13 (above) and MMP3 (below) in TNFα-stimulated CH at day 1, 3 and 6 analyzed by Western blot. Data (*n* Effect of TNFα on MMPs protein expression in CH. PDH and expressed as relative values (CTR = 1). Statistical analysis was performed by paired *t*-test. Significance vs. appropriate CTR for each time point are shown as * $p < 0.05$.

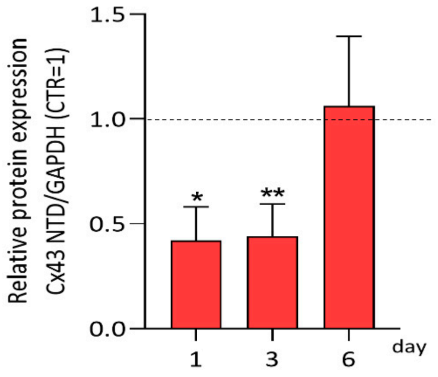


**Supplementary Figure S3.** Western blot analysis of the expression of Cx43 using an Ab raised against an epitope at the NTD in TNFα-stimulated CH at day 1, 3 and 6. Data (*n* Western blot analysis of the expression of Cx43 using an Ab raised against an epitope at the NTD in TNFα-stimulat. CTR is shown as * $p < 0.05$ and ** $p < 0.01$.

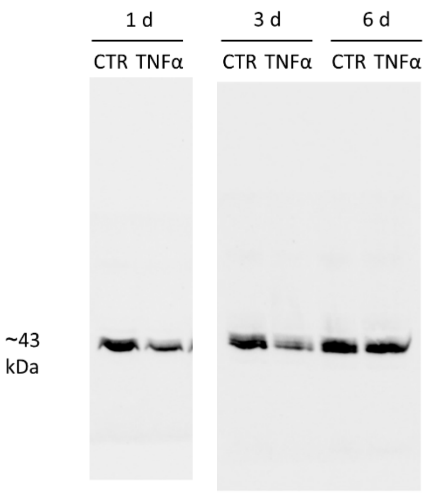


**Supplementary Figure S4.** Full Western blot image of Figure 1b. Cx43 expression was detected by an antibody raised against CTD.

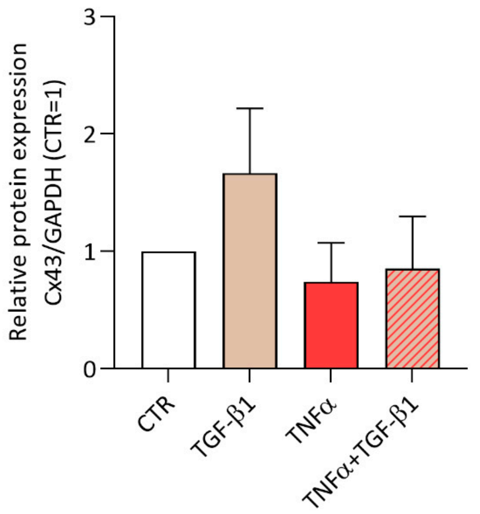


**Supplementary Figure S5**. Quantification of the expression of Cx43 in CH treated with TNFα or/and TGFβ at day 6 analyzed by Western blot. Data (*n* = 6 independent experiments) were normalized on GAPDH and expressed as relative values (CTR = 1).

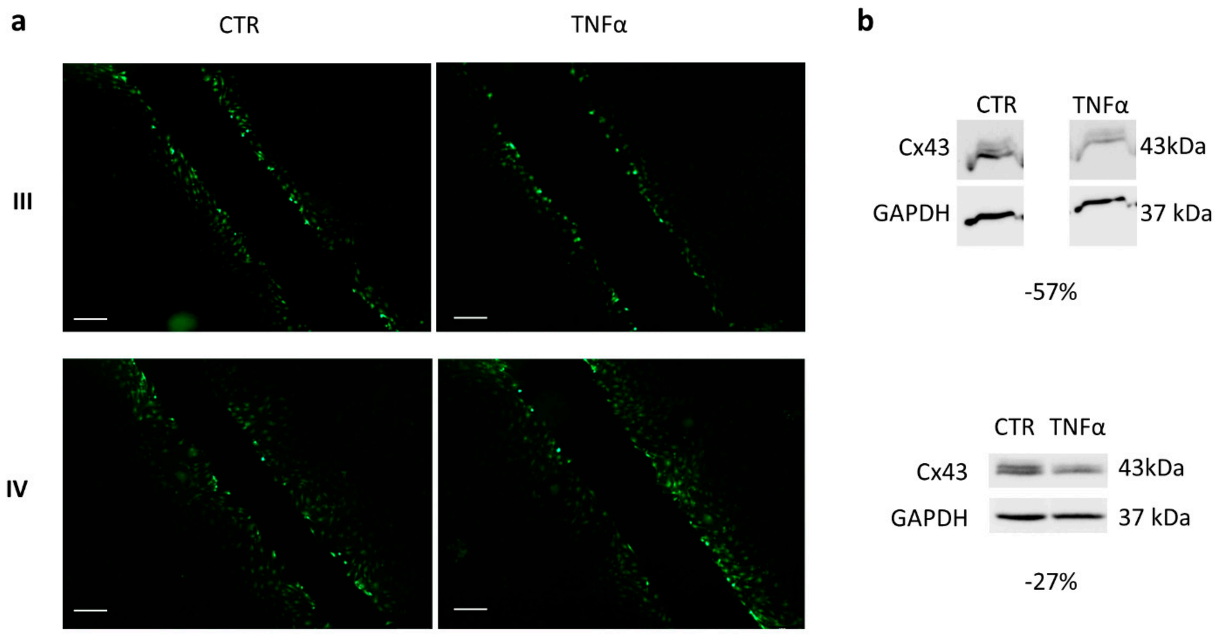


**Supplementary Figure S6**. (**a**) Illustrative images of scrape loading/dye transfer (SL/DT) assay in CTR (**on the left**) and TNFα-treated (**on the right**) CH, at day 3. Scale bars: 200 µm. (**b**) Immunoblot of each experiment is reported. The percentage of decrease in Cx43 protein expression in TNFα-treated CH compared to CTR is shown.

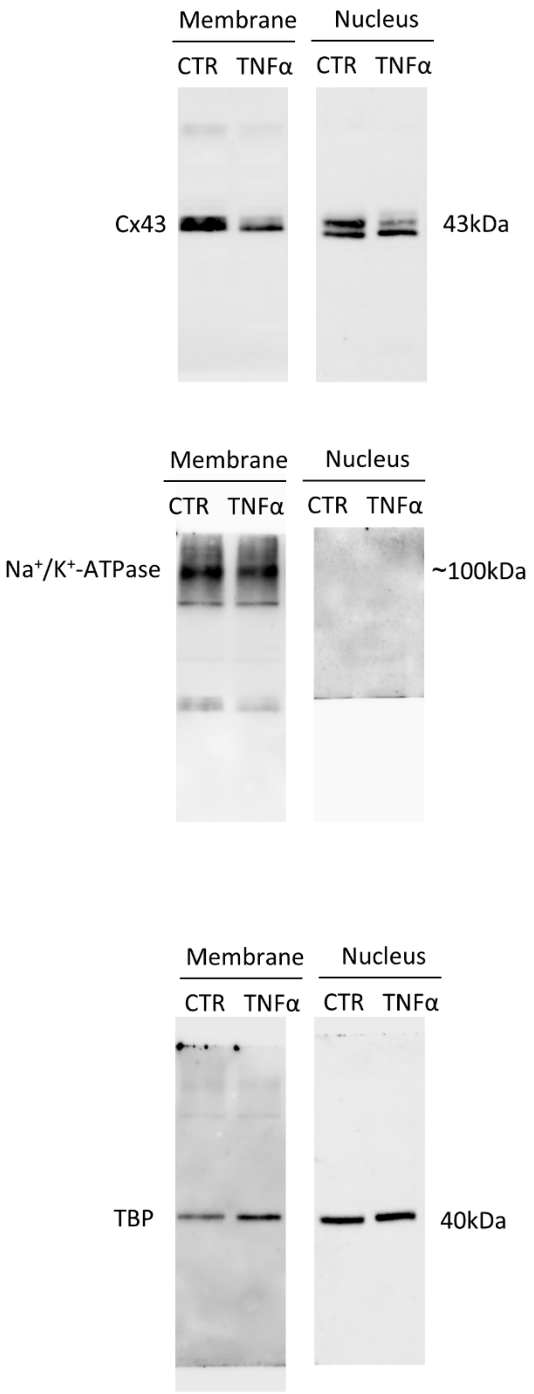


**Supplementary Figure S7**. Full Western blot image of Figure 5b.
